# Patients with melanoma treated with immune checkpoint inhibitors who had non-thyroid endocrine and skin immune-related adverse events have better prognosis: A systematic review and meta-analysis

**DOI:** 10.3389/fonc.2022.976224

**Published:** 2022-09-14

**Authors:** Qian Sun, Hongyan Sun, Nan Wu, Yue Hu, Fangqing Zhang, Xianling Cong

**Affiliations:** ^1^ Department of Dermatology, China-Japan Union Hospital of Jilin University, Changchun, China; ^2^ Department of Biobank, China-Japan Union Hospital of Jilin University, Changchun, China

**Keywords:** immune checkpoint inhibitors, immune-related adverse events, melanoma, prognosis, meta-analysis, survival

## Abstract

**Background:**

Several studies have reported an association between the occurrence of immune-related adverse events (irAEs) and prognosis in patients with melanoma treated with immune checkpoint inhibitors (ICIs), but the results remain controversial. We conducted a systematic review and meta-analysis to investigate the association between irAEs and survival in patients with melanoma treated with ICIs.

**Methods:**

We searched the PubMed, Web of Science, and China National Knowledge Infrastructure databases through May 5, 2022 for clinical studies evaluating the association between irAEs and in melanoma patients treated with ICIs. Combined hazard ratios (HRs) for overall survival (OS) and progression-free survival (PFS) were calculated using fixed- or random-effects models based on heterogeneity.

**Results:**

A total of 60 articles were included, with 16,520 patients. In patients with melanoma treated with ICIs, the occurrence of irAEs was significantly associated with better OS (HR, 0.58; 95% confidence interval [CI], 0.51–0.66; *P*<0.00001) and PFS (HR, 0.61; 95%CI, 0.51–0.72; *P*<0.00001). Endocrine irAEs (OS, HR, 0.81; 95%CI, 0.72–0.92; *P*=0.001; PFS: HR, 0.84; 95%CI, 0.73–0.96, *P*=0.009), skin irAEs (OS, HR, 0.59; 95%CI, 0.41–0.85; *P*=0.004; PFS: HR, 0.43; 95%CI, 0.36–0.52; *P*<0.00001), vitiligo (OS, HR, 0.22; 95%CI, 0.15–0.31; *P*<0.00001; PFS, HR, 0.33; 95%CI, 0.25–0.44; *P*<0.00001), and grade 1–2 irAEs (OS, HR, 0.67; 95%CI, 0.58–0.78; *P*<0.00001; PFS, HR, 0.62; 95%CI, 0.51–0.76; *P*<0.00001) showed similar results. However, thyroid, lung, gastrointestinal, liver, and grade 3–4 irAEs were not significantly associated with OS and PFS. The occurrence of non-thyroid endocrine irAEs was significantly associated with better OS (HR, 0.22; 95%CI, 0.15–0.31; *P*<0.00001). In patients with melanoma treated with anti-programmed cell death protein 1 (OS, HR, 0.61; 95%CI, 0.51–0.72; *P*<0.00001; PFS, HR, 0.59; 95%CI, 0.47–0.74; *P*<0.00001), the association between irAEs and clinical benefit was clearer than in patients treated with anti-cytotoxic T-lymphocyte-associated protein 4 (OS, HR, 0.68; 95%CI, 0.52–0.89; *P*=0.005; PFS, HR, 0.93; 95%CI, 0.49–1.78; *P*=0.83).

**Conclusion:**

Among patients with melanoma treated with ICIs, those who developed non-thyroid endocrine irAEs and cutaneous irAEs have better prognosis. This suggests that non-thyroid endocrine irAEs and cutaneous irAEs may be a prognostic biomarker for patients with melanoma treated with ICIs.

**Systematic review registration:**

https://www.crd.york.ac.uk/PROSPERO/, identifier CRD42022338308.

## Introduction

Melanoma is highly malignant and occurs mostly in the skin, accounting for more than 75% of the deaths related to skin cancer (1). Patients in the early stages can be treated with surgery, but those in the later stages have a poor prognosis and cannot be cured with chemotherapy (2, 3). Recently, the treatment of advanced melanoma has significantly improved, owing to advances in immunotherapy with immune checkpoint inhibitors (ICIs) (4–6). The prominent members of such drugs include ipilimumab, a cytotoxic T-lymphocyte-associated protein 4 (CTLA-4) inhibitor; nivolumab and pembrolizumab, anti-programmed cell death protein 1 (PD-1) agents; and atezolizumab, durvalumab, and avelumab, which are anti-programmed cell death 1 ligand 1(PD-L1) agents (7). ICIs have been widely used in the treatment of melanoma, non-small cell lung cancer, renal cancer, other solid tumors, and hematologic malignancies, not only in metastatic but also in adjuvant settings (6–10).

Despite their clinical efficacy, ICIs may cause immunotoxicity due to a highly active immune response, which is known as immune-related adverse event (irAE). ICI-associated irAEs can potentially involve multiple organs or systems, including the skin (e.g., rash, vitiligo), gastrointestinal system (e.g., diarrhea, colitis), endocrine system (e.g., thyroid irAEs, hypophysitis), liver (e.g., hepatitis), and lung (e.g., pneumonitis) (11, 12). A key question is whether there is an association between the occurrence of irAEs and outcomes of patients treated with ICIs. Recent studies have observed an association between irAEs and prognosis; however, the findings are controversial. The relationship between irAEs and survival remains inconclusive (13–18).

Previous meta-analyses mostly focused on examining the relationship between irAEs and efficacy, whereas few studies explored the relationship between irAEs and prognosis, and these studies mainly focused on patients with all types of cancer (19–23). Therefore, to determine whether the occurrence of irAEs following ICI use in patients with melanoma is associated with prognosis, we systematically reviewed and analyzed the literature to determine whether irAEs can be used as a biomarker to predict prognosis in patients with melanoma treated with ICIs, providing a better understanding and insight into individualized therapy.

## Material and methods

This systematic review and meta-analysis followed the Preferred Reporting Items for Systematic Reviews and Meta-analyses (PRISMA) guidelines (24). This study is registered on PROSPERO, number CRD42022338308.

### Search strategy

PubMed, Web of Science, and China National Knowledge Infrastructure databases were searched to identify relevant articles from inception through May 5, 2022. The terms used in the search strategy were “Immune related adverse events OR irAE OR treatment related adverse events OR TRAE OR adverse events OR AE” (all fields) AND “melanoma” (all fields) AND “prognosis OR prognostic OR survival OR outcome” (all fields). here were no restrictions on publication year, while the language was restricted to English and Chinese. Titles, abstracts, and articles with these keywords were screened by two independent reviewers using pre-established inclusion criteria to identify relevant studies for inclusion in the systematic review. Moreover, we reviewed the reference lists of the retrieved studies and recent reviews for potentially related studies.

### Inclusion and exclusion criteria

The inclusion criteria were as follows: (1) published as original articles; (2) evaluated patients with melanoma who received CTLA-4, PD-1, or PD-L1 inhibitor monotherapy or in combination; (3) reported the associations between irAEs and outcomes (overall survival [OS] or progression-free survival [PFS]) in patients with melanoma treated with ICIs; (4) provided sufficient data to estimate the hazard ratio (HR) and 95% confidence intervals (CIs) of OS or PFS in patients who developed irAEs of any type, endocrine (any, thyroid, non- thyroid), pulmonary, skin (any or vitiligo), gastrointestinal (any, colitis, diarrhea), and hepatic; (5) published in English; and (6) had the most informative or recent publication if studies were obtained from the same institution with a duplicate study population.

The exclusion criteria were as follows: (1) did not provide available data to estimate HRs and 95%CIs for OS or PFS; (2) had duplicate data or overlapping study populations; (3) published in reviews, case reports, editorial letters, conferences, comments, or responses; (4) had concomitant treatment with other experimental agents; and (5) ICIs was used as an adjunctive therapy.

### Data extraction and quality assessment

Data were extracted by two authors from the identified studies in duplicate using a common extraction form. Differences were resolved by consensus.

The following information was extracted from the included studies: first author, publication year, study design, country, follow-up time, number of patients treated with ICIs, Melanoma subtype, ICI types, irAE types and grades, observational outcomes, HRs, and corresponding 95% CIs of OS or PFS in patients with different irAE types or grades. Several studies have provided both multivariate and univariate HRs, and we selected the former. When the articles did not provide HRs and 95% CIs but only survival curves, we extracted the data from Kaplan-Meier curves by digitizing the curves using the open-source Engauge Digitizer software (http://digitizer.sourceforge.net/) and estimating the univariate HR (25).

The quality of each study was independently assessed by two researchers using the Newcastle–Ottawa Quality Assessment Scale (NOS) (26). Scores ranged from 0 to 9 for quality assessment, and studies with scores ≥6 were rated high quality.

### Statistical analysis

Pooled HRs and 95% CIs were calculated to assess the association between the occurrence of irAEs and OS and PFS. HR >1 indicated a poor prognosis, while HR <1 indicated a good prognosis. If the study reported HR and 95% CI, we extracted them directly. If HR was not reported directly in the article, we used the data extracted from the survival curves to calculate log HR and related standard errors using a spreadsheet provided by Tierney et al. (27). The data from Kaplan–Meier survival curves were read by two independent researchers using Engauge Digitizer version 4.1 to reduce variability, and the specific method refers to the description of Zhou et al. (25).

A pooled analysis of all reports was performed using the inverse variance method. Forest plots were drawn to summarize information from individual studies and the pooled effect sizes of the study objects. The chi-square test and I^2^ were used to evaluate the heterogeneity between the included studies (28). I^2^ values ≤25% indicated no heterogeneity, ≤50% indicated minimal heterogeneity, ≤75% indicated moderate heterogeneity, and >75% indicated significant heterogeneity. Statistical methods for meta-analysis included fixed- and random-effects models. The analysis model was selected according to heterogeneity. I^2^ >50% indicated the presence of heterogeneity, and a random-effects model was applied to partially eliminate the effects of heterogeneity. Otherwise, a fixed-effects model was used (29). Subgroup analyses were performed to assess the effect of these variables on the results based on the study design, geographical area, ICI type and melanoma subtype. Publication bias was assessed using funnel plots and Begg’s and Egger’s tests (30, 31). A sensitivity analysis was performed by omitting one study at a time to evaluate the potential bias and robustness of the overall risk estimate.

All analyses were performed using Review Manager version 5.4.1 (The Cochrane Collaboration, Copenhagen: The Nordic Cochrane Centre, 2020) and Stata version 17.0 (Stata Corporation, College Station, TX, USA). A *P*-value <0.05 indicated statistical significance.

## Results

### Study characteristics

**Figure 1:**
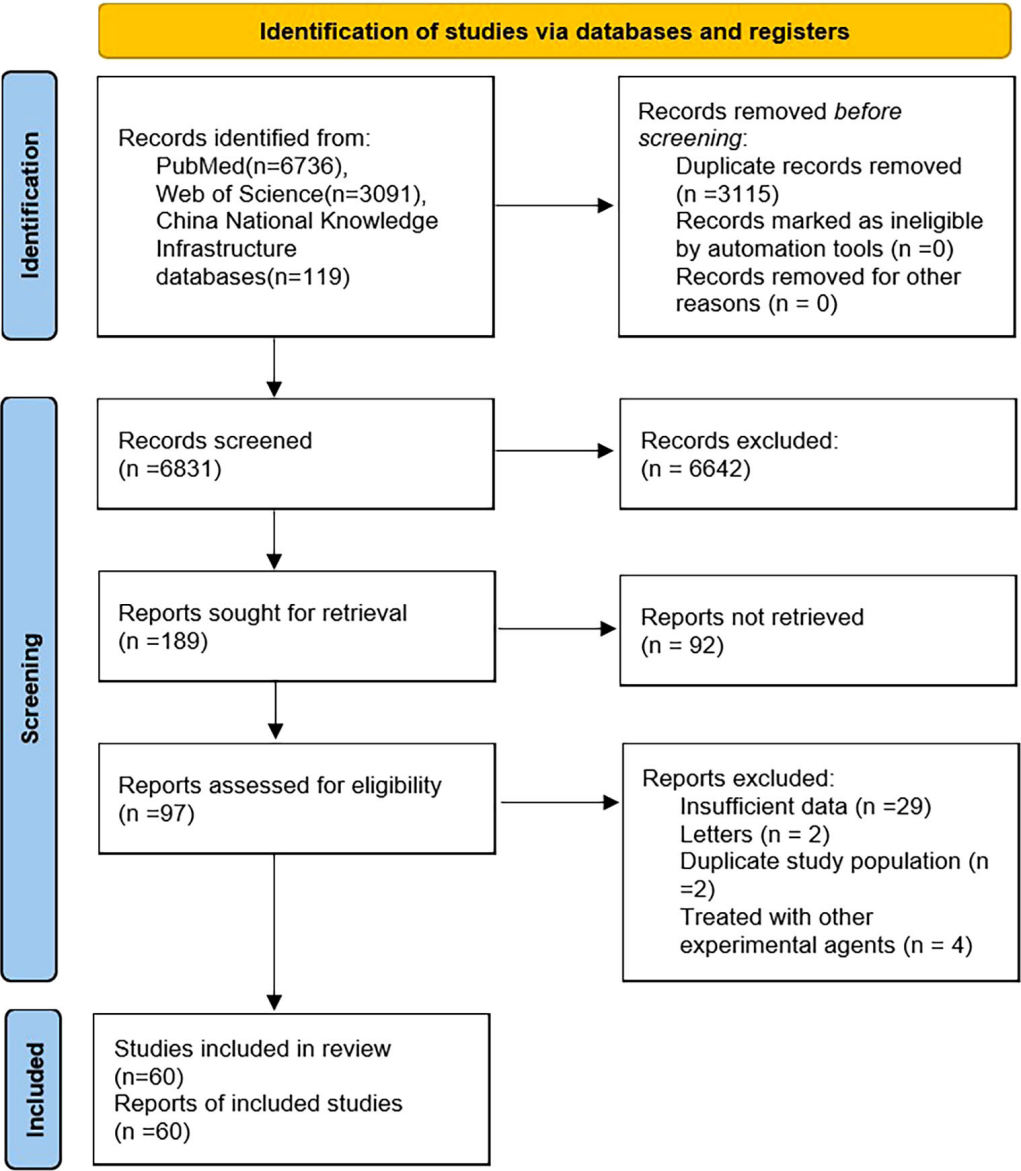
Flowchart of study selection process.

Using the search strategy described above, 9946 citations were retrieved. After screening the titles, abstracts, publication types, and full texts of each article, 97 articles were identified that investigated the correlation between irAEs and prognosis of patients with melanoma. Of these studies, 37 were excluded, including 29 that did not have sufficient data, two that were letters, two that had overlapping study populations, and four that treated with other experimental agents. Finally, 60 articles were included in the meta-analysis (13–18, 32–85) (**Figure 1**).

Of the included studies, 46 were retrospective (13–17, 32, 34, 35, 39, 41, 43–52, 54, 55, 57, 59–72, 74–78, 82–85), 13 were prospective (18, 33, 36–38, 40, 53, 56, 58, 73, 79–81) (including four studies that extracted data from clinical trials (18, 33, 73, 79)), and one included both retrospective and prospective data (42). All included studies assessed the relationship between the presence of irAEs and prognosis (12 of them examined the effect of irAE grades (14, 37, 45, 57, 58, 68, 71, 73, 76, 82, 84, 85)), 18 studies assessed endocrine irAEs (any, thyroiditis, or hypophysitis) (32, 34, 44, 46–48, 51, 55, 56, 60, 68–70, 73, 76, 78, 81, 85), five assessed pulmonary irAEs (32, 34, 46, 56, 76), 21 assessed skin irAEs (any, rash, or vitiligo) (32, 34, 38, 40, 42, 46–48, 51, 53, 54, 56, 62–64, 73, 74, 76, 79, 81, 85), nine assessed gastrointestinal irAEs (any, colitis, or diarrhea) (32, 34, 46, 56, 58, 73, 76, 77, 85), and five assessed hepatic irAEs (32, 34, 36, 76, 85). A total of 57 studies included OS data (13–18, 32–39, 41–58, 60–71, 73–85) and 34 included PFS data (14, 15, 18, 32, 33, 36–42, 47, 49, 54, 55, 58–60, 62, 64, 66, 68–72, 75, 76, 78, 79, 81, 82, 85). Geographically, 18 studies were from Asian countries (35, 39, 48, 56, 61–66, 69, 76–81, 85), 24 from European countries (13–16, 33, 36–38, 41–43, 45, 47, 50, 53–55, 58, 59, 68, 74, 75, 82, 84), six from the United States (32, 44, 46, 52, 67, 70), five from Canada (17, 51, 57, 71, 83), one from the United States and Canada (73), two from Australia (40, 60), one from the United States and Australia (34), and one from the United States and Australia and Germany (49). Moreover, two studies pooled data from multiple countries internationally (18, 72). A total of 16,520 patients (14–2,561 patients per study) were included in this meta-analysis. The details of the 60 studies are presented in **Table 1**.

**Table 1:** The detailed characteristics of the included studies.

First author, publication year	Study design	Country	Follow-up time	Patients treated with ICIs	Melanoma subtype	ICIs types	Included irAEs types^a^	Included irAEs grades^b^	Included outcome	NOS score
Abu-Sbeih, 2019	Retrospective	USA	Mean 1.7 years	346	/	Anti-PD-1 or anti-CTLA-4 or combination	Endocrine, Hepatic, Pulmonary, Skin, Gastrointestinal	Any	OS, PFS	7
Arheden, 2019	Retrospective	Sweden	Median 17 months	116	Cutaneous	Anti-PD-1	Any	Any	OS	8
Ascierto, 2014	Clinic trial	Italy	Median 6.7 months	855	Cutaneous, ocular or mucosal	Anti-PD-1	Any	Any	OS	7
Bai, 2021	Retrospective	US, Australia	Median 206 weeks	947	Cutaneous, acral, mucosal, ocular	Anti-PD-1	Any, Endocrine, Pulmonary, Skin, Gastrointestinal, Hepatic	Any	OS, PFS	8
Ben-Betzalel, 2019	Retrospective	Israel	Median15 months	144	/	Anti-PD-1	Any	Any	OS	7
Biewenga, 2021	Prospective	Netherlands	Median 6.5 months	2561	Exclude uveal	Anti-PD-1 or anti-CTLA-4 or combination	Hepatitis	Any	OS, PFS	9
Bisschop, 2019	Prospective	Netherlands	Median 37 months	147	Cutaneous, mucosal	Anti-PD-1	Any	Low, high	OS, PFS	8
Bottlaender, 2020	Prospective	France	/	189	/	Anti-PD-1	Skin, Rush, Vitiligo	Any	OS, PFS (skin)	6
Byun, 2021	Retrospective	South Korea	Median 13.7 months	134	Acral, mucosal, uveal	Anti-PD-1 or anti-CTLA-4 or combination	Any	Any	OS, PFS	7
Chan, 2020	Prospective	Australia	Median 40.7 months	82	/	Anti-PD-1	Skin	Any	PFS	7
Cybulska-Stopa, 2021	Retrospective	Poland	Median 13.6 months	585	Cutaneous, mucosal	Anti-PD-1	Any	Any, Grade 1-2, Grade 3-4	OS, PFS	7
Dick, 2016	Retrospective	Germany	/	86	Cutaneous, choroidea, mucosal	Anti-CTLA-4	Any	Any	OS, PFS	6
Dimitriou, 2021	Retrospective	Switzerland	Median 27 months	256	Cutaneous, mucosal, uveal	Anti-PD-1 or anti-CTLA-4 or combination	Any	Any	OS, PFS	8
Dousset, 2021	Retrospective and prospective	France	Median 16 months	457	Cutaneous, mucosal, choroid	Anti-PD-1	Vitiligo	Any	OS, PFS	7
Dupont, 2020	Retrospective	France	Median 24 months	120	Cutaneous	Anti-PD-1	Any	Any	OS	7
Faje, 2018	Retrospective	USA	/	281	/	Anti-CTLA-4	Hypophysitis	Any	OS	7
Farolfi, 2012	Retrospective	Italy	/	36	Cutaneous, mucosal, choroid	Anti-CTLA-4	Any	Grade>2	OS	6
Freeman-Keller, 2016	Retrospective	American	median 139 weeks	148	/	Anti-PD-1	Any, Vitiligo, Hypothyroidism, Hyperthyroidism, Pneumonitis, Rash, Gastrointestinal	Any	OS	9
Frelau, 2021	Retrospective	France	Median 32.8 months	110	/	Anti-PD-1 or anti-PD-1 + anti-CTLA-4	Thyroid, Vitiligo	Any	OS, PFS	8
Fujisawa, 2018	Retrospective	Japan	/	60	Cutaneous, mucosal	Anti-PD-1 + anti-CTLA-4	Any, Endocrine, Skin	Any	OS	6
Ghisoni, 2021	Retrospective	Switzerland	/	220	/	Anti-PD-1 or anti-CTLA-4 or combination or anti-PD-L1	Any	Any	OS	6
Halle, 2021	Retrospective	USA, Australia, Germany	Median 25.1 months	62	/	Anti-PD-1 or anti-CTLA-4 or anti-PD-L1	Any	Any	OS, PFS	6
Hernando-Calvo, 2021	Retrospective	Spain	Median 14.4 months	52	/	Anti-PD-1 or anti-CTLA-4	Any	Any	OS	6
Holstead, 2021	Retrospective	Canada	/	87	Cutaneous, mucosal, uveal	Anti-PD-1	Any, Endocrine (Thyroid), Skin	Any	OS	7
Horvat, 2015	Retrospective	USA	/	298	/	Anti-CTLA-4	Any	Any	OS	6
Hua, 2016	Prospective	France	Median 441 days	67	Cutaneous, mucosal	Anti-PD-1	Vitiligo	Any	OS	7
Indini, 2019	Retrospective	Italy	Median 9 months	173	Cutaneous, mucosal, uveal	Anti-PD-1	Any, Vitiligo	Any	OS, PFS(skin)	7
Karhapaa, 2022	Retrospective	Finland	Median 23.5 months	140	/	Anti-PD-1 or anti-CTLA-4 or combination	Endocrine	Any	OS, PFS	7
Kobayashi, 2020	Prospective	Japan	343±319 days	66	/	Anti-PD-1 or anti-CTLA-4 or anti-PD-L1	Any, Endocrine, Pituitary, Thyroid, Pulmonary, Skin, Gastrointestinal	Any	OS	6
Ksienski, 2021	Retrospective	Canada	Median 19.8 months	95	Cutaneous, mucosal, ocular	Anti-PD-1 + anti-CTLA-4	Any	Any, Grade 3-4	OS	8
Ksienski, 2022	Retrospective	Canada	Median 29.9 months	302	Cutaneous, mucosal, ocular	Anti-PD-1 + anti-CTLA-4	Any	Any	OS	7
Lang, 2019	Prospective	Germany	Median 12.6 months	100	Cutaneous, ocular	Anti-CTLA-4	Colitis, Diarrhea	Any, Grade 3 (Diarrhea)	OS, PFS	6
Mesti, 2021	Retrospective	Slovenia	/	99	/	Anti-PD-1 or anti-CTLA-4 or combination	Any	Any	PFS	6
Muir, 2021	Retrospective	Australia	Median 11.3 months	1246	/	Anti-PD-1 or anti-CTLA-4 or combination or anti-PD-L1	Thyroid, Thyrotoxicosis	Any	OS, PFS	7
Muto, 2019	Retrospective	Japan	/	30	Cutaneous, mucosal, uveal, conjunctival	Anti-PD-1 + anti-CTLA-4	Any	Any	OS	7
Nakamura, 2017	Retrospective	Japan	/	35	/	Anti-PD-1	Vitiligo	Any	OS, PFS	6
Nakano, 2020	Retrospective	Japan	Median 646 days	128	Cutaneous, mucosal, ocular	Anti-PD-1	Skin, Vitiligo	Any	OS	8
Namikawa, 2020	Retrospective	Japan	Median 15 months	14	Uveal	Anti-PD-1	Vitiligo	Any	OS, PFS	7
Okada, 2019	Retrospective	Japan	Median 470 days	15	/	Anti-PD-1	Any	Any	OS	7
Otsuka, 2020	Retrospective	Japan	Median 509 days	27	Mucosal	Anti-PD-1	Any	Any	OS, PFS	7
Robert, 2021	Three clinical trials	Australia, Austria, Belgium, Canada, Chile, Colombia, France, Germany, Israel, Netherlands, New Zealand, Norway, Spain, Sweden, UK, USA, Switzerland, Italy, Norway, Argentina	Median 42.4 months	1567	/	Anti-PD-1	Any	Any	OS, PFS	7
Rose, 2020	Retrospective	US	/	68	/	Anti-PD-1 or anti-CTLA-4 or combination or anti-PD-L1	Any	Any	OS	6
Sakakida, 2019	Retrospective	Japan	Median 29 weeks	26	/	Anti-PD-1	Thyroid	Any	OS, PFS	6
Serna-Higuita, 2021	Retrospective	Germany	Median 24 months	319	Cutaneous, ocular	Anti-PD-1 or anti-PD-1 + anti-CTLA-4	Any, Endocrine	Grade 1-2 (OS), Grade 3-4	OS, PFS	8
Snyders, 2019	Retrospective	USA	Median 52.7 months	117	/	Anti-CTLA-4	Hypophysitis	Any	OS, PFS	8
Suo, 2020	Retrospective	Canada	Median 24 months	186	Cutaneous, mucosal, ocular	Anti-PD-1	Any	Any, Grade 3-4	OS, PFS	7
Sznol, 2017	Retrospective	Australia, Europe, Israel, New Zealand,US, France	Median 13.2 months	448	Cutaneous, mucosal, ocular	Anti-PD-1 + anti-CTLA-4	Any	Any	PFS	6
Tarhini, 2021	Phase III trial	US, Canada	Median 57.4 months	1034	Cutaneous	Anti-CTLA-4	Any, Endocrine, Rash, Gastrointestinal	Any, Grade 1-2, Grade 3-4	OS	7
Villa-Crespo, 2022	Retrospective	Spain	at least three months	153	Cutaneous, mucosal	Anti-PD-1 or anti-CTLA-4 or combination	Any, Skin	Any	OS	9
Wei, 2019	Retrospective	UK	Median 7 months	51	Cutaneous, ocular	Anti-PD-1 or anti-CTLA-4 or combination	Any	Any	OS, PFS	7
Wu, 2020	Retrospective	China	Median 9.1 months	49	Cutaneous, mucosal	Anti-PD-1	Any, Endocrine, Pulmonary, Skin, Vitiligo, Colitis, Hepatic	Grade 1-2, Grade 3-5	OS, PFS	7
Yamada, 2021	Retrospective	Japan	/	149	/	Anti-PD-1 or anti-CTLA-4 or combination or anti-PD-L1	Gastrointestinal	Any	OS	7
Yamauchi, 2019	Retrospective	Japan	Median 359 days	42	/	Anti-PD-1	Thyroid	Any	OS, PFS	8
Yamazaki, 2017	Phase II study	Japan	Median 18.8 months	24	Cutaneous	Anti-PD-1	Vitiligo	Any	OS, PFS	7
Yamazaki, 2020	Prospective	Japan		547	Cutaneous, mucosal, ocular, uveal	Anti-CTLA-4	Any	Any	OS	7
			12 months							
Yamazaki, 2021	Prospective	Japan	/	124	Cutaneous, mucosal	Anti-PD-1	Any, Thyroid, Skin, Vitiligo	Any	OS, PFS	7
Ye, 2021	Retrospective	UK	Median 18.3 months	354	/	Anti-PD-1 or anti-PD-1 + anti-CTLA-4	Any	Any, Grade 1-2, Grade 3-4 (OS), Any (PFS)	OS, PFS	6
Yeung, 2021	Retrospective	Canada	/	143	/	Anti-PD-1 or anti-CTLA-4	Any	Any	OS	7
Yousaf, 2015	Retrospective	UK	/	110	/	Anti-CTLA-4	Any	Grade 3-4	OS	7
Zhao, 2020	Retrospective	China	/	93	Cutaneous, mucosal, uveal	Anti-PD-1	Any, Endocrine, Skin, Hepatic, Gastrointestinal	Any, Grade 1-2, Grade 3-4	OS, PFS	8

^a^"Any" means that irAEs of any type are included in the statistics; ^b^"Any" refers to the fact that there is no classification of grades in the original text or irAEs of any grades are included in the statistics.

Abbreviations: irAEs, immune-related adverse events; OS, overall survival; PFS, progression free survival; ICIs, immune checkpoint inhibitors; NOS, the Newcastle-Ottawa Quality Assessment Scale; PD-1, programmed cell death protein 1; CTLA-4, cytotoxic T-lymphocyte-associated protein 4; PD-L1, programmed cell death 1 ligand 1.

The quality of each study included in our meta-analysis was evaluated according to NOS, with a score ranging from 6 to 9 (7.0 points on average) (**Table 1**). All studies were considered adequate for inclusion in this meta-analysis.

### Pooled analyses

#### Correlation of irAEs with OS and PFS

**Figure 2:**
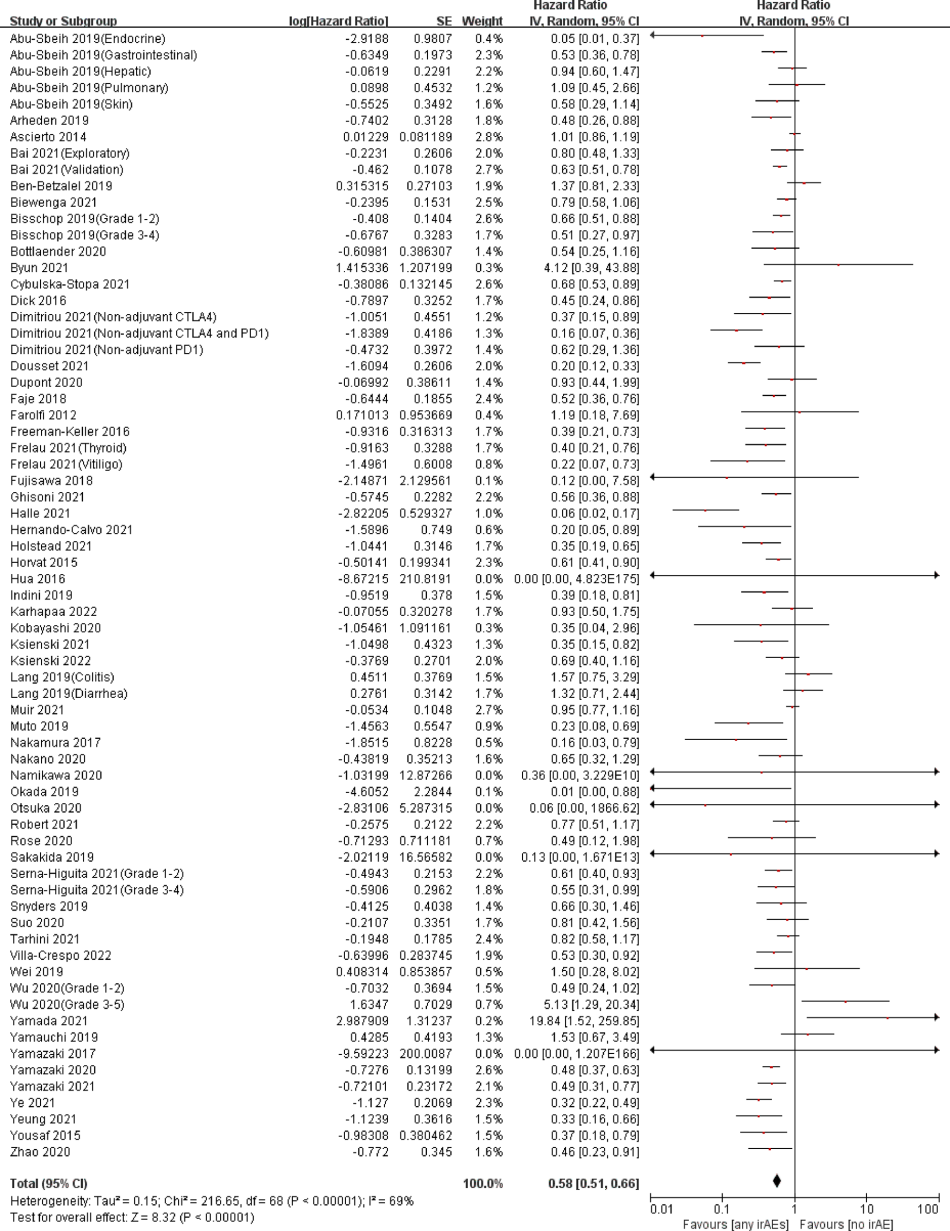
Forest plot of the association between irAEs and OS in patients with melanoma treated with ICIs.

**Figure 3:**
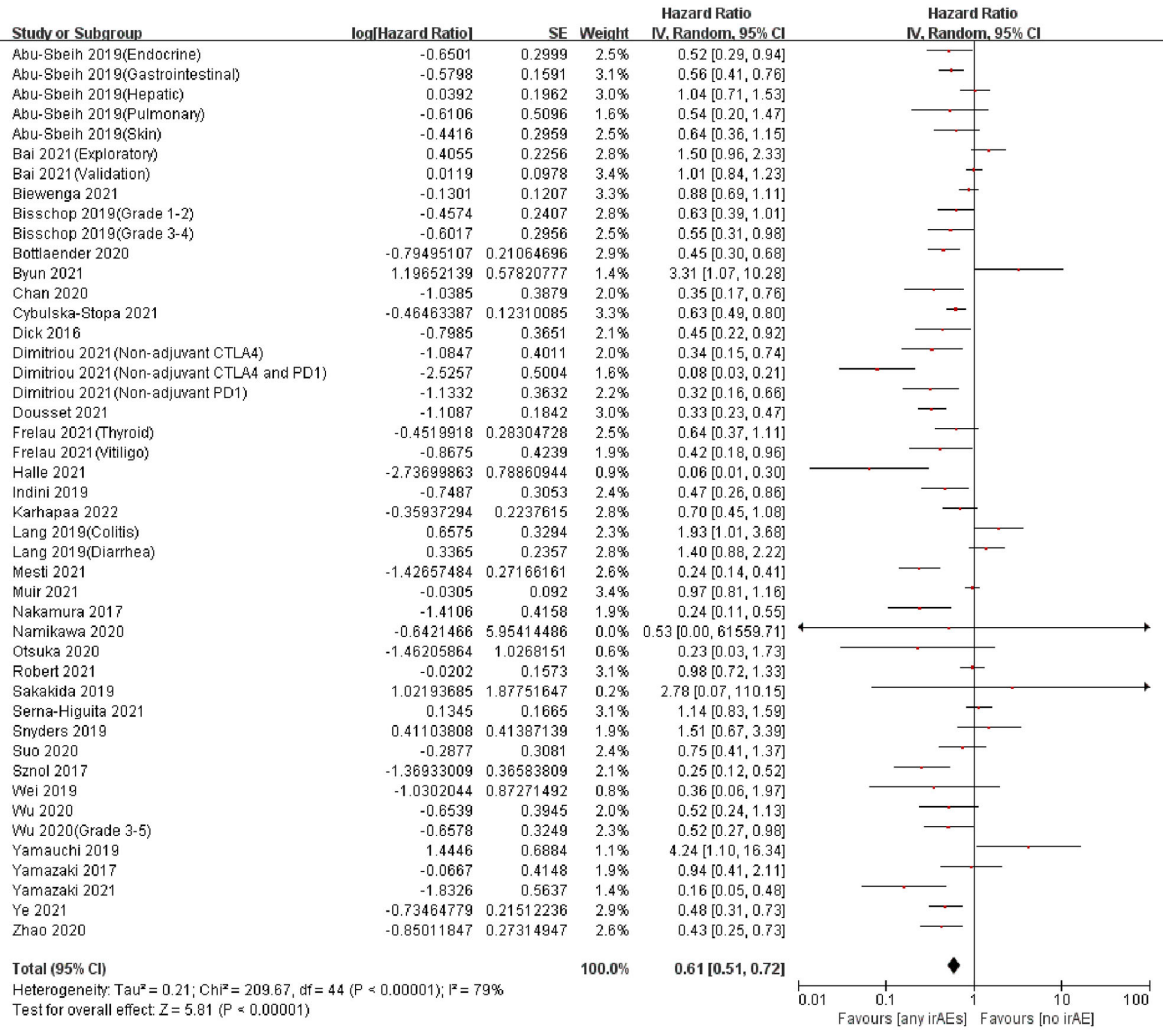
Forest plot of the association between any irAEs and PFS in patients with melanoma treated with ICIs.

A total of 57 articles assessed the association between the occurrence of irAEs and OS in patients with melanoma (13–18, 32–39, 41–58, 60–71, 73–75, 77–86), and 34 assessed PFS (14, 15, 18, 32, 34, 36–42, 47, 49, 54, 55, 58–60, 62, 64, 66, 68–72, 75, 76, 78, 79, 81, 82, 85). Among these, the study of Abu-Sbeih et al. contained OS and PFS data of five types of irAEs (32); Bai et al.’s study contained OS and PFS data of two cohorts (exploratory and validation) (34); the studies of Bisschop et al. (37) and Wu et al. (76) contained OS and PFS data of two different irAE grades; the studies of Frelau et al. (47) and Lang et al. (58) included OS and PFS data of two types of irAEs; Serna-Higuita et al.’s (68) study included OS data of two different irAE grades; and Dimitriou et al.’s (41) study included data of three different treatment cohorts. Pooled analysis showed that among patients with melanoma treated with ICIs, those who had irAEs had a significant advantage in OS (pooled HR, 0.58; 95%CI, 0.51–0.66; *P <*0.00001; I^2 ^= 69%) (**Figure 2**) and PFS (pooled HR, 0.61; 95%CI, 0.51–0.72; *P <*0.00001; I^2 ^= 79%) (**Figure 3**) compared with patients who did not have irAEs (**Table 2**).

**Table 2:** Summary analysis of the relationship between immune-related adverse events and survival in patients with melanoma.

Subset	OS					PFS				
	Studies (n)	Patients with/without irAEs	Hazard Risk (95% CI)	P-value	I^2^(%)	Studies (n)	Patients with/without irAEs	Hazard Risk (95% CI)	P-value	I^2^(%)
**Any irAE**	69	5946/8288	0.58(0.51–0.66)	<0.00001	69	45	3232/5888	0.61(0.51–0.72)	<0.00001	79
Grade 1-2	7	872/870	0.67(0.58–0.78)	<0.00001	0	4	247/560	0.62(0.51–0.76)	<0.00001	0
Grade 3-4	12	722/1266	0.81(0.52–1.26)	0.35	71	7	234/926	1.43(0.86–2.37)	0.16	72
**Endocrine**	18	1275/3171	0.81(0.72–0.92)	0.001	49	12	815/1862	0.84(0.73–0.96)	0.009	32
Thyroid	8	622/1237	1.07(0.64–1.81)	0.79	63	5	597/914	0.91(0.77–1.07)	0.24	10
Non-thyroid	3	88/357	0.55(0.40–0.76)	0.0004	0	1	15/97	1.51(0.67–3.39)	0.3184	/
**Pulmonary**	5	36/728	1.71(0.61–4.79)	0.31	62	3	28/536	1.37(0.40–4.64)	0.61	72
**Skin**	21	1093/2378	0.59(0.41–0.85)	0.004	68	12	312/1291	0.43(0.36–0.52)	<0.00001	50
Vitiligo	12	205/1113	0.22(0.15–0.31)	<0.00001	0	7	123/628	0.33(0.25–0.44)	<0.00001	7
**Gastrointestinal**	10	876/1342	0.94(0.67–1.30)	0.69	55	6	281/576	1.10(0.68–1.77)	0.70	73
**Hepatic**	7	196/1575	0.86(0.69–1.08)	0.19	49	7	196/1575	0.97(0.81–1.16)	0.75	20

irAEs, immune-related adverse events; OS, overall survival; PFS, progression free survival.

**Figure 4:**
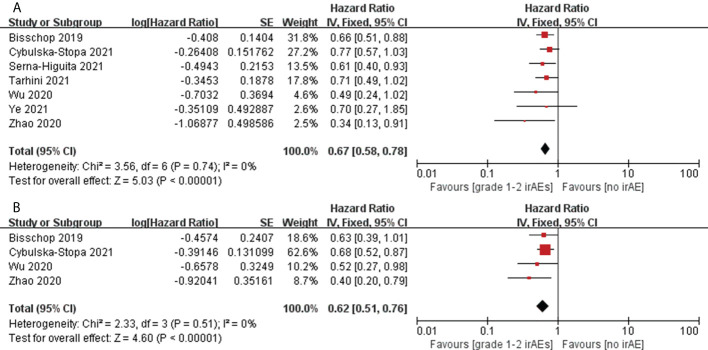
Forest plots of the association between grade1-2 irAEs and OS **(A)** and PFS **(B)** in patients with melanoma treated with ICIs.

Twelve articles assessed the association between patients with melanoma with grade 3–4 irAEs and OS (14, 37, 45, 57, 58, 68, 71, 73, 76, 82, 84, 85), and seven articles assessed patients with melanoma with grade 1–2 irAEs (14, 37, 68, 73, 76, 82, 85). Seven articles assessed the association between patients with melanoma with grade 3–4 irAEs and PFS (14, 37, 58, 68, 71, 76, 85), and four articles assessed patients with melanoma with grade 1–2 irAEs (14, 37, 76, 85). However, among melanoma patients treated with ICIs, patients who had grade 1–2 irAEs had a significant advantage in OS (pooled HR, 0.67; 95%CI, 0.58–0.78; *P <*0.00001; I^2 ^= 0%) and PFS (pooled HR, 0.62; 95%CI, 0.51–0.76; *P <*0.00001; I^2 ^= 0%) compared with patients who did not have irAEs (**Figure 4**), while patients who had grade 3–4 irAEs had no significant difference in OS (pooled HR, 0.81; 95%CI, 0.52–1.26; *P*=0.35; I^2 ^= 71%) and PFS (pooled HR, 1.43; 95%CI, 0.86–2.37; *P*=0.16; I^2 ^= 72%) (**Supplementary Figure 1**) (**Table 2**).

#### Correlation of endocrine irAEs with OS and PFS

**Figure 5:**
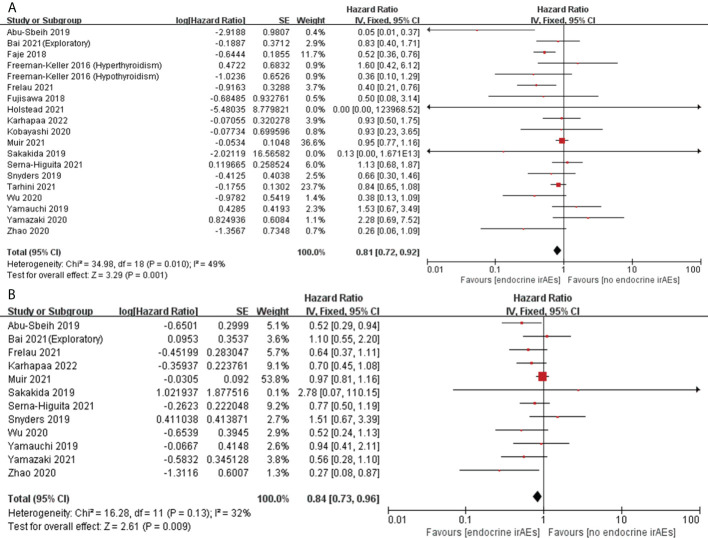
Forest plots of the association between endocrine irAEs and OS **(A)** and PFS **(B)** in patients with melanoma treated with ICIs.

**Figure 6:**
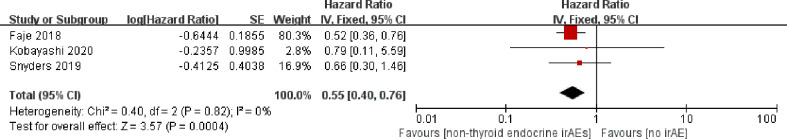
Forest plots of the association between non-thyroid endocrine irAEs and OS in patients with melanoma treated with ICIs.

Eighteen articles assessed the association between the occurrence of endocrine irAEs and OS in patients with melanoma (32, 34, 44, 46–48, 51, 55, 56, 60, 68–70, 73, 76, 78, 81, 85), and 12 also assessed the association with PFS (32, 34, 47, 55, 60, 68–70, 76, 78, 81, 85). Pooled analysis showed that, among patients with melanoma treated with ICIs, patients who developed endocrine irAEs had a significant advantage in OS (pooled HR, 0.81; 95%CI, 0.72–0.92; *P*=0.001; I^2 ^= 49%) and PFS (pooled HR, 0.84; 95%CI, 0.73–0.96; *P*=0.009; I^2 ^= 32%) compared with patients who did not develop endocrine irAEs (**Figure 5**). Moreover, a separate pooled analysis was performed on thyroid irAEs, which had a higher incidence. Seven articles assessed the association between the occurrence of thyroid irAEs and OS in patients with melanoma (46, 47, 56, 60, 69, 78, 81) (one of which assessed only hyperthyroidism and hypothyroidism (46)), and five assessed the association with PFS (47, 60, 69, 78, 81). Interestingly, pooled analysis showed that thyroid irAEs were not significantly associated with OS (pooled HR, 1.07; 95%CI, 0.64–1.81; *P*=0.79; I^2 ^= 63%) and PFS (pooled HR, 0.91; 95%CI, 0.77–1.07; *P*=0.24; I^2 ^= 10%) (**Supplementary Figure 2**). This suggests that the effect of endocrine toxicity on prognosis may be limited to non-thyroid toxicity. Therefore, we further analyzed the association between non-thyroid irAEs and survival. Three articles evaluated the association between the occurrence of non-thyroid irAEs (pituitary) and OS in melanoma patients (44, 56, 70). The pooled results showed that patients with non-thyroid irAEs had a significant advantage in OS (pooled HR 0.55; 95% CI 0.40–0.76; *P*=0.0004; I^2 ^= 0%) (**Figure 6**). However, only one article evaluated the association between the occurrence of non-thyroid irAEs (hypophysitis) and PFS in melanoma patients (70), and the results showed no significant association between them (HR 1.51; 95%CI 0.67–3.39; *P* = 0.3184) (**Table 2**).

#### Correlation of pulmonary irAEs with OS and PFS

Five articles assessed the association between the occurrence of pulmonary irAEs and OS in patients with melanoma (32, 34, 46, 56, 76), and three assessed the association with PFS (32, 34, 76). Pooled analysis showed that the incidence of pulmonary irAEs was not significantly associated with OS (pooled HR, 1.71; 95%CI, 0.61–4.79; *P*=0.31; I^2 ^= 62%) and PFS (pooled HR, 1.37; 95%CI, 0.40–4.64; *P*=0.61; I^2 ^= 72%) in patients with melanoma treated with ICIs (**Table 2**, **Supplementary Figure 3**).

#### Correlation of skin irAEs with OS and PFS

**Figure 7:**
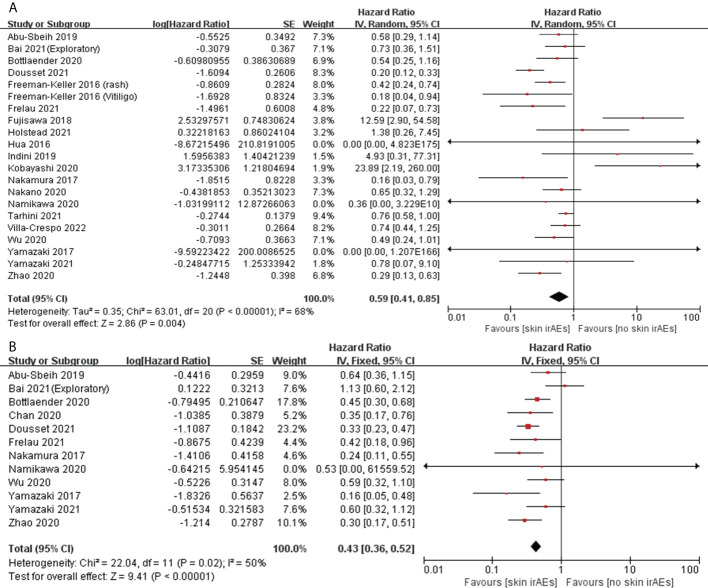
Forest plots of the association between skin irAEs and OS **(A)** and PFS **(B)** in patients with melanoma treated with ICIs.

**Figure 8:**
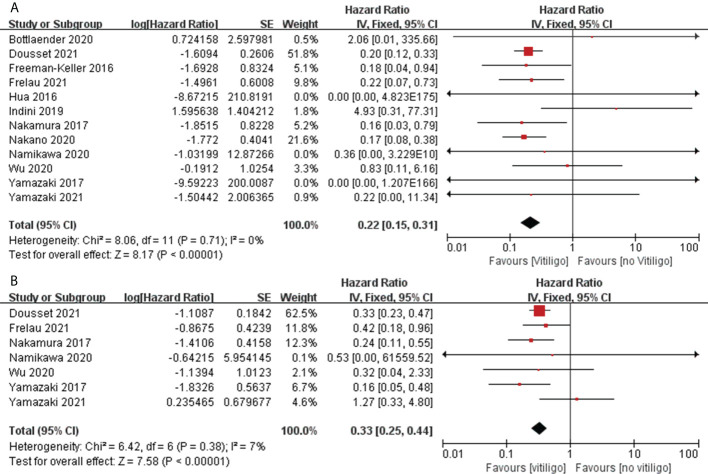
Forest plots of the association between vitiligo and OS **(A)** and PFS **(B)** in patients with melanoma treated with ICIs.

Twenty studies assessed the association between the occurrence of skin irAEs and OS in patients with melanoma (32, 34, 38, 42, 46–48, 51, 53, 54, 56, 62–64, 73, 74, 76, 79, 81, 85), while12 assessed the association with PFS (32, 34, 38, 42, 47, 54, 62, 64, 76, 79, 81, 85). Pooled analysis showed that, among patients with melanoma treated with ICIs, those who had skin irAEs had a significant advantage in OS (pooled HR, 0.59; 95%CI, 0.41–0.85; *P* = 0.004; I^2^ = 68%) and PFS (pooled HR, 0.43; 95%CI, 0.36–0.52; *P <*0.00001; I^2^ = 50%) compared with patients who did not have skin irAEs (**Figure 7**). Furthermore, we performed a pooled analysis of vitiligo cases with a high incidence of skin irAEs. Twelve studies assessed the association between the occurrence of vitiligo and OS in patients with melanoma (38, 42, 46, 47, 53, 54, 62–64, 76, 79, 81), and seven assessed the association with PFS (42, 47, 62, 64, 76, 79, 81). Pooled analysis showed that patients with vitiligo had a significant advantage in OS (pooled HR, 0.22; 95%CI, 0.15–0.31; *P <*0.00001; I^2 ^= 0%) and PFS (pooled HR, 0.33; 95%CI, 0.25–0.44; *P <*0.00001; I^2 ^= 7%) compared with patients with melanoma without vitiligo (**Figure 8**) (**Table 2**).

#### Correlation of gastrointestinal irAEs with OS and PFS

Nine articles assessed the association between the occurrence of gastrointestinal irAEs and OS in patients with melanoma (32, 34, 46, 56, 58, 73, 76, 77, 85), and five assessed the association with PFS (32, 34, 58, 76, 85). Among these, one assessed colitis and diarrhea only (58), and one assessed colitis only (76). Pooled analysis showed that the occurrence of gastrointestinal irAEs was not significantly associated with OS (pooled HR, 0.94; 95%CI, 0.67–1.30; *P*=0.69; I^2 ^= 55%) and PFS (pooled HR, 1.10; 95%CI, 0.68–1.77; *P*=0.70; I^2 ^= 73%) in patients with melanoma treated with ICIs (**Table 2**, **Supplementary Figure 4**).

#### Correlation of hepatic irAEs with OS and PFS

Five articles assessed the association between the occurrence of hepatic irAEs and OS and PFS in patients with melanoma (32, 34, 36, 76, 85). The study by Bai et al. (34) included data from two cohorts, and the study by Wu et al. (76) contained data of two different irAE grades. Pooled analysis showed that the occurrence of hepatic irAEs was not significantly associated with OS (pooled HR, 0.86; 95%CI, 0.69–1.08; *P*=0.19; I^2^ = 49%) and PFS (pooled HR, 0.97; 95%CI, 0.81–1.16; *P*=0.75; I^2^ = 20%) in patients with melanoma treated with ICIs (**Table 2**, **Supplementary Figure 5**).

### Subgroup analysis

We performed a subgroup analysis of the association of irAEs with OS and PFS based on the study design, geographic region, ICI type, and melanoma subtype to assess the impact of these variables on the results (**Table 3**).

**Table 3:** Subgroup analyses of the association between immune-related adverse events and survival.

Groups	OS					PFS				
	Studies (n)	Patients with/without irAEs	Hazard Risk (95% CI)	P-value	I^2^(%)	Studies (n)	Patients with/without irAEs	Hazard Risk (95% CI)	P-value	I^2^(%)
**Study design**										
Retrospective	54	3878/5847	0.55(0.48-0.64)	<0.00001	63	31	2571/4078	0.56(0.46-0.69)	<0.00001	78
Prospective	14	1985/2085	0.74(0.60-0.91)	0.004	66	13	578/1454	0.77(0.58-1.01)	0.06	76
Retrospective and prospective	1	83/356	0.2(0.12-0.33)	0.00001	/	1	83/356	0.33(0.23-0.47)	<0.00001	/
**Geographic area**										
Asia	19	763/852	0.68(0.46-1.02)	0.06	62	11	205/330	0.62(0.38-1.03)	0.07	67
Europe	28	1761/2962	0.55(0.44-0.68)	<0.00001	74	20	1194/2349	0.54(0.42-0.69)	<0.00001	81
America	16	2120/2483	0.58(0.48-0.70)	<0.00001	42	7	421/1608	0.73(0.55-0.96)	0.02	44
Others	6	1302/1991	0.63(0.45-0.88)	0.007	84	7	1412/1601	0.73(0.52-1.03)	0.07	83
**ICI type**										
Anti-PD-1	31	1849/3001	0.61(0.51-0.72)	<0.00001	52	23	1452/2379	0.59(0.47-0.74)	<0.00001	75
Anti-CTLA-4	12	1996/1488	0.68(0.52-0.89)	0.005	76	5	150/266	0.93(0.49-1.78)	0.83	79
Anti-PD-1 + anti-CTLA-4	5	249/85	0.23(0.14-0.38)	<0.00001	0	4	324/168	0.22(0.03-1.43)	0.11	90
Anti-PD-1/L1 or anti-CTLA-4	4	116/85	0.19(0.06-0.55)	0.002	54	/	/	/	/	/
Anti-PD-1/L1 or anti-CTLA-4 or combination	14	1428/3065	0.71(0.55-0.92)	0.008	63	11	1056/2647	0.61(0.46-0.81)	0.0006	78
Anti-PD-1 or anti-PD-1 + anti-CTLA-4	4	210/451	0.51(0.37-0.69)	<0.0001	6	3	152/315	0.74(0.41-1.31)	0.3	71
**Melanoma subtype**										
Ocular melanoma excluded	14	1596/1318	0.67(0.57-0.79)	<0.00001	25	8	406/994	0.62(0.46-0.84)	0.002	69
Ocular melanoma included	26	2203/2701	0.58(0.45-0.73)	<0.00001	72	18	1456/1509	0.62(0.44-0.88)	0.007	85
Unknown	29	2147/4269	0.54(0.43-0.68)	<0.00001	72	19	1370/3385	0.58(0.46-0.74)	<0.00001	75

ICIs, immune checkpoint inhibitors; PD-1/L1, programmed cell death protein 1/ligand 1; CTLA-4, cytotoxic T-lymphocyte-associated protein 4; OS, overall survival; PFS, progression free survival.

#### Stratification based on study design

We divided the included literatures into retrospective studies, prospective studies (including clinical trials), and others. The results of subgroup analysis showed that, whether retrospectively or prospectively, patients with melanoma who had irAEs had better OS than patients who did not have irAEs (retrospective, pooled HR, 0.55; 95%CI, 0.48–0.64; *P*<0.00001; I^2^ = 63%; prospective, pooled HR, 0.74; 95%CI, 0.60–0.91; *P*=0.004; I^2 ^= 66%) (**Supplementary Figure 6**). The advantage of PFS was only observed in retrospective studies (pooled HR, 0.56; 95%CI, 0.46–0.69; *P*<0.00001; I^2 ^= 78%), but there was no statistical difference in prospective studies (pooled HR, 0.77; 95%CI, 0.58–1.01; *P*=0.06; I^2 ^= 76%) (**Supplementary Figure 7**).

#### Stratification based on geographic region

Subgroup analysis showed that patients with melanoma who had irAEs had a significant advantage in OS (Europe, pooled HR, 0.55; 95%CI, 0.44–0.68; *P*<0.00001; I^2 ^= 74%; Americas, pooled HR, 0.58; 95%CI, 0.48–0.70; *P*<0.00001; I^2 ^= 42%) and PFS (Europe, pooled HR, 0.54; 95%CI, 0.42–0.69; *P*<0.00001; I^2 ^= 81%; Americas, pooled HR, 0.73; 95%CI, 0.55–0.96; *P*=0.02; I^2 ^= 44%) in Europe and Americas, but there was no significant difference in Asia (**Supplementary Figures 8, 9**).

#### Stratification based on the type of ICIs

CTLA-4 and PD-1 are two different pathways that downregulate T-cell function, and their clinical efficacy and safety may vary according to their different mechanisms. Therefore, we conducted a subgroup analysis of these two types of ICIs. The results showed that irAEs were statistically associated with better OS (pooled HR, 0.61; 95%CI, 0.51–0.72; *P*<0.00001; I^2 ^= 52%) and PFS in patients with melanoma who received anti-PD-1 therapy alone (pooled HR, 0.59; 95%CI, 0.47–0.74; *P*<0.00001; I^2 ^= 75%). Of the patients with melanoma treated with anti-CTLA-4 only, irAEs were statistically associated with better OS (pooled HR, 0.68; 95%CI, 0.52–0.89; *P*=0.005; I^2 ^= 76%) but not with PFS (pooled HR, 0.93; 95%CI, 0.49–1.78; *P*=0.83; I^2 ^= 79%). Similarly, in patients treated with combination of anti-PD1 and anti-CTLA-4, irAEs were associated with better OS (pooled HR, 0.23; 95%CI, 0.14–0.38; *P*<0.00001; I^2 ^= 0%), but not with PFS (pooled HR, 0.22; 95%CI, 0.03–1.43; *P*=0.11; I^2 ^= 90%) (**Supplementary Figures 10, 11**).

#### Stratification based on melanoma subtype

We analyzed the association between any irAEs and survival in study populations with and without ocular melanoma. The results showed that irAEs was associated with better OS (Ocular melanoma excluded, pooled HR, 0.67; 95%CI, 0.57–0.79; *P*<0.00001; I^2 ^= 25%; Ocular melanoma included, pooled HR, 0.58; 95%CI, 0.45–0.73; *P*<0.00001; I^2 ^= 72%) and PFS (Ocular melanoma excluded, pooled HR, 0.62; 95%CI, 0.46–0.84; *P*=0.002; I^2 ^= 69%; Ocular melanoma included, pooled HR, 0.62; 95%CI, 0.44–0.88; *P*=0.007; I^2 ^= 85%) regardless of whether uveal melanoma was included (**Supplementary Figures 12, 13**).

### Sensitivity analysis

Sensitivity analyses investigated the impact of a single study on the overall risk estimates by omitting one study in each turn to demonstrate that most overall risk estimates were not significantly modified by any single study. In studies of associations between irAEs and OS and PFS, the overall estimate remained stable after any studies were sequentially deleted. The studies by Abu-Sbeih et al. (32) and Yamada et al. (77) may contribute to the moderate heterogeneity of OS outcomes in patients with gastrointestinal irAEs (I^2 ^= 55%), and heterogeneity was significantly reduced when these two studies were omitted sequentially (I^2 ^= 36% and I^2 ^= 45%, respectively). The study by Abu-Sbeih et al. (32) had a significant impact on PFS in patients with gastrointestinal irAEs. After excluding this study, the pooled HR changed from 1.10 (95%CI, 0.68–1.77) to 1.39 (95%CI, 1.03–1.88), and heterogeneity significantly decreased (I^2 ^= 73% to I^2 ^= 0%). The exploratory study by Bai et al. (34) may contribute to the minimal heterogeneity of OS outcomes in patients with hepatic irAEs (I^2 ^= 49%), and the heterogeneity was significantly reduced when this study was omitted (I^2 ^= 13%). The studies by Muir et al. (60) and Zhao et al. (85) may contribute to the minimal heterogeneity of OS outcomes in patients with hepatic irAEs (I^2 ^= 32%), which was significantly reduced when the two studies were omitted sequentially (I^2 ^= 8% and I^2 ^= 21%, respectively). The study of Muir et al. (60) had a significant effect on PFS outcome in patients with thyroid irAEs, and after excluding this study, the pooled HR changed from 0.91 (95%CI, 0.77–1.07; I^2 ^= 10%) to 0.67 (95%CI, 0.46–0.98; I^2 ^= 0%). The study by Wu et al. (76) may contribute to moderate heterogeneity of OS and PFS outcomes in patients with pulmonary irAEs (I^2 ^= 62% and I^2 ^= 72%, respectively), which was significantly reduced when this study was omitted (I^2 ^= 30% and I^2 ^= 0%, respectively). The exploratory study by Bai et al. (34) may contribute to the minimal heterogeneity of PFS outcomes in patients with skin irAEs (I^2 ^= 50%), and the heterogeneity was significantly reduced after the omission of this study (I^2 ^= 20%).

### Publication bias

**Figure 9:**
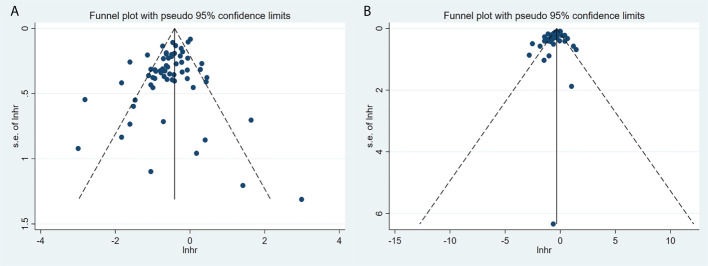
The funnel plot of the association of any irAEs with OS **(A)** and PFS **(B)**.

Egger’s test showed possible publication bias in the analysis of the relationship between the presence of irAEs and OS and PFS in patients with melanoma (both *P*=0.011), whereas Begg’s test showed the opposite result (*P*=0.331 and *P*=0.193, respectively) (the funnel plots are shown in **Figure 9**). Analysis of the relationship between any grade 1–2 irAE and OS in patients with melanoma showed possible publication bias by both Begg’s and Egger’s tests (*P*=0.044 and *P*=0.035, respectively). For the rest, using Begg’s and Egger’s tests, there was no evidence of publication bias for grade 3–4, lung, skin, vitiligo, gastrointestinal, hepatic, endocrine, thyroid, and non-thyroid endocrine irAEs in relation to melanoma survival (all *P*>0.05).

## Discussion

Identifying reliable biomarkers of response to ICIs has been the focus of intensive research since the initiation of ICI use in the treatment of malignancies (87). Early clinical observations in the era of novel immunotherapeutics have hinted at a potential association between irAEs and patient survival, but the results remain controversial (23, 32, 51, 57, 71). Previous studies have focused on the association between the occurrence of irAEs and efficacy and survival in all patients with cancer or lung cancer (19, 88, 89), while there was a lack of systematic review on the association between the occurrence of irAEs and survival in patients with melanoma.

To the best of our knowledge, this is the first systematic review and meta-analysis to date to demonstrate the association between the occurrence of irAEs and survival outcomes in patients with melanoma using ICIs. Sixty studies were eventually included in this meta-analysis, including a total of 16,520 patients. Pooled analysis showed that patients with melanoma who received ICIs and had irAEs had significantly better OS and PFS than patients without irAEs. The lack of statistical significance in OS and PFS among patients with melanoma with grade 3 and above irAEs may be due to the increased mortality associated with higher side effects or glucocorticoid therapy. Our conclusions are consistent with previously published meta-analyses of all patients with cancer, suggesting a positive association between the occurrence of irAEs and PFS and OS, and the occurrence of irAEs may be a prognostic factor for patients with cancer treated with ICIs (19–23, 86). Several studies have also found that the occurrence of low-grade irAEs, but not severe irAEs, is associated with better ICI outcomes in patients with cancer (19, 23, 88). Similarly, Zhao et al. found that the presence of irAEs was strongly associated with better survival and response in patients with non-small cell lung cancer receiving anti-PD-1 therapy, suggesting its potential role as a predictive biomarker of outcomes after PD-1 inhibition (89).

Interestingly, all subgroups stratified by study design and type of ICIs showed consistent results with the overall estimate for improvement in OS, but no statistically significant differences were observed for PFS in the prospective study group, the anti-CTLA-4 monotherapy group, or the anti-PD-1 and anti-CTLA-4 combination group. The specific mechanism underlying this discrepancy remains unclear, although it might be partially influenced by differences in ICI location (90). The sparse data of the CTLA-4 subgroup deserves further study, and a large prospective cohort study with well-designed and confounding controls is required to generate further insights. A subgroup analysis stratified by geographic region showed that the presence of irAEs was not significantly associated with either OS or PFS in Asian patients with melanoma. Although the underlying biological mechanism of differential prognosis is unclear, melanoma subtypes, genetic susceptibility, and environmental factors may play a role (91).

Furthermore, we investigated the correlation between the different types of irAEs and survival outcomes. Our pooled analysis showed that patients with melanoma who had endocrine irAEs or vitiligo had better OS and PFS. Patients with melanoma who had cutaneous irAEs had better OS, while PFS was not statistically significant. However, thyroid, pulmonary, gastrointestinal, and hepatic irAEs were not significantly associated with the OS or PFS. This suggested that the effect of endocrine irAEs on prognosis may be limited to non-thyroid irAEs. Therefore, we further analyzed the association between non-thyroid irAEs and survival. The summary results of three related literatures showed that non-thyroid irAEs was associated with better OS. However, only one article reported the effect of non-thyroid irAEs (Hypophysitis) on PFS, and the results suggested that it was not significantly associated with PFS. Due to the small number of literatures that can be included in this analysis, the results are sensitive. Zhou et al. (23) and Fan et al. (19) also observed a survival benefit in patients with irAEs in those with endocrine and dermatological abnormalities but not in those with gastrointestinal, pulmonary, or hepatic abnormalities. Wang et al. (88) found that the occurrence of irAEs (especially skin, endocrine, and gastrointestinal irAEs) in patients with lung cancer was significantly associated with better OS and was a predictor of enhanced ICI efficacy. However, we have to admit that vitiligo frequently appears late in the course of treatment, which will inevitably have an impact on the results. Due to the limitations of the original data, we were unable to exclude vitiligo from the overall analysis. Therefore, a large number of prospective studies are needed to evaluate the impact of non-vitiligo irAEs on the prognosis of melanoma patients treated with ICIs.

Our study is the largest systematic review and meta-analysis to date on the association between the occurrence of irAEs and survival outcomes in patients with melanoma treated with ICIs. This study gathered all available studies to date, including retrospective and prospective studies. In addition to any irAEs, the risk factors considered in our analysis included different grades and types of irAEs (endocrine, skin, gastrointestinal, pulmonary, and hepatic), making our analysis more comprehensive, specific, and detailed. Another advantage of this meta-analysis is that it provides an analysis of the two main clinical outcomes of OS and PFS and conducts subgroup analysis based on study design, geographic region, and type of ICIs to minimize the impact of potential confounding factors on the results and more truly reflect the impact of target risk factors on the results.

While the assessment scores for quality indicated that most included studies were of high quality, we acknowledge that this meta-analysis has some limitations; therefore, the results should be interpreted with caution. Limitations of this study include the following (1): When the original study did not provide HR, the Kaplan–Meier survival curve was used to extract data, and HR was calculated using a spreadsheet, which inevitably caused small statistical error. (2) While a portion of HRs were derived from multivariate models, these studies did not consistently adjust for potential risk factors. Some HRs were not adjusted for multivariable factors or calculated directly from the survival curve of the studies, so it is uncertain whether the observed results are responses only to the target risk factors and not to other confounding factors. (3) Both prospective and retrospective studies were included in this meta-analysis. There is a possibility of recall bias in retrospective studies. (4) There is great heterogeneity among different studies. This may be due to differences in baseline characteristics among the eligible studies, such as the number of participants, geographic area, type of melanoma, treatment, study design, irAE definition, and outcome measurements. Although subgroup analyses in study design, ICI type, and geographic area were used to find the source of heterogeneity to minimize the impact of these factors on survival, moderate to significant heterogeneity was detected, and the effects of heterogeneity were partially eliminated using a random-effects model. (5) Egger’s test found significant publication bias in the combined results of OS and PFS in patients with irAEs, indicating that the combined analysis results may be exaggerated. (6) Another potential confounding factor is the treatment of toxicity. Patients with high-grade irAEs may have been treated with glucocorticoids, which may have a negative impact on survival, but this part of data is difficult to obtain. Therefore, more rigorous cohort studies are needed to confirm the results of this study.

In summary, this is the first meta-analysis to assess the effects of irAEs on the outcomes of patients with melanoma treated with ICIs. Our study supports the idea that the development of non-thyroid endocrine irAEs and cutaneous irAEs can improve the prognosis of patients with melanoma treated with ICIs. Further studies are needed to understand the pathophysiology of irAEs and identify prognostic biomarkers that can specifically predict immunosuppressant treatment response.

## Conclusion

Our meta-analysis shows that, among patients with melanoma treated with ICIs, those who developed non-thyroid endocrine irAEs and cutaneous irAEs have better prognosis, suggesting that developed non-thyroid endocrine irAEs and cutaneous irAEs may be a prognostic biomarker of ICI treatment for patients with melanoma.

## Data availability statement

The original contributions presented in the study are included in the article/**Supplementary Material**. Further inquiries can be directed to the corresponding author.

## Author contributions

Conceptualization, supervision: XC and QS. Data extraction: QS and YH. Writing – original draft preparation: HS. Methodology, software: QS and HS. Writing – reviewing and editing: FZ, HS, and NW. All authors contributed to the article and approved the submitted version.

## Funding

This study was supported by the Science and technology development project of Jilin Province (20210204150YY, 20200601010JC, 20200201598JC) and the Special Project of Health Scientific Research talents in Jilin Province (2020SC239).

## Conflict of interest

The authors declare that the research was conducted in the absence of any commercial or financial relationships that could be construed as a potential conflict of interest.

## Publisher’s note

All claims expressed in this article are solely those of the authors and do not necessarily represent those of their affiliated organizations, or those of the publisher, the editors and the reviewers. Any product that may be evaluated in this article, or claim that may be made by its manufacturer, is not guaranteed or endorsed by the publisher.
