# Supplementary material for: Patients with melanoma treated with immune checkpoint inhibitors who had non-thyroid endocrine and skin immune-related adverse events have better prognosis: A systematic review and meta-analysis
Source: Front Oncol. 2022 Sep 14;12:976224. doi: 10.3389/fonc.2022.976224 (PMC9515964; doi:10.3389/fonc.2022.976224)
Supplement: Supplementary file 1 [file DataSheet_1.docx]

Supplementary Material

# Supplementary Figures and Tables

## Supplementary Figures


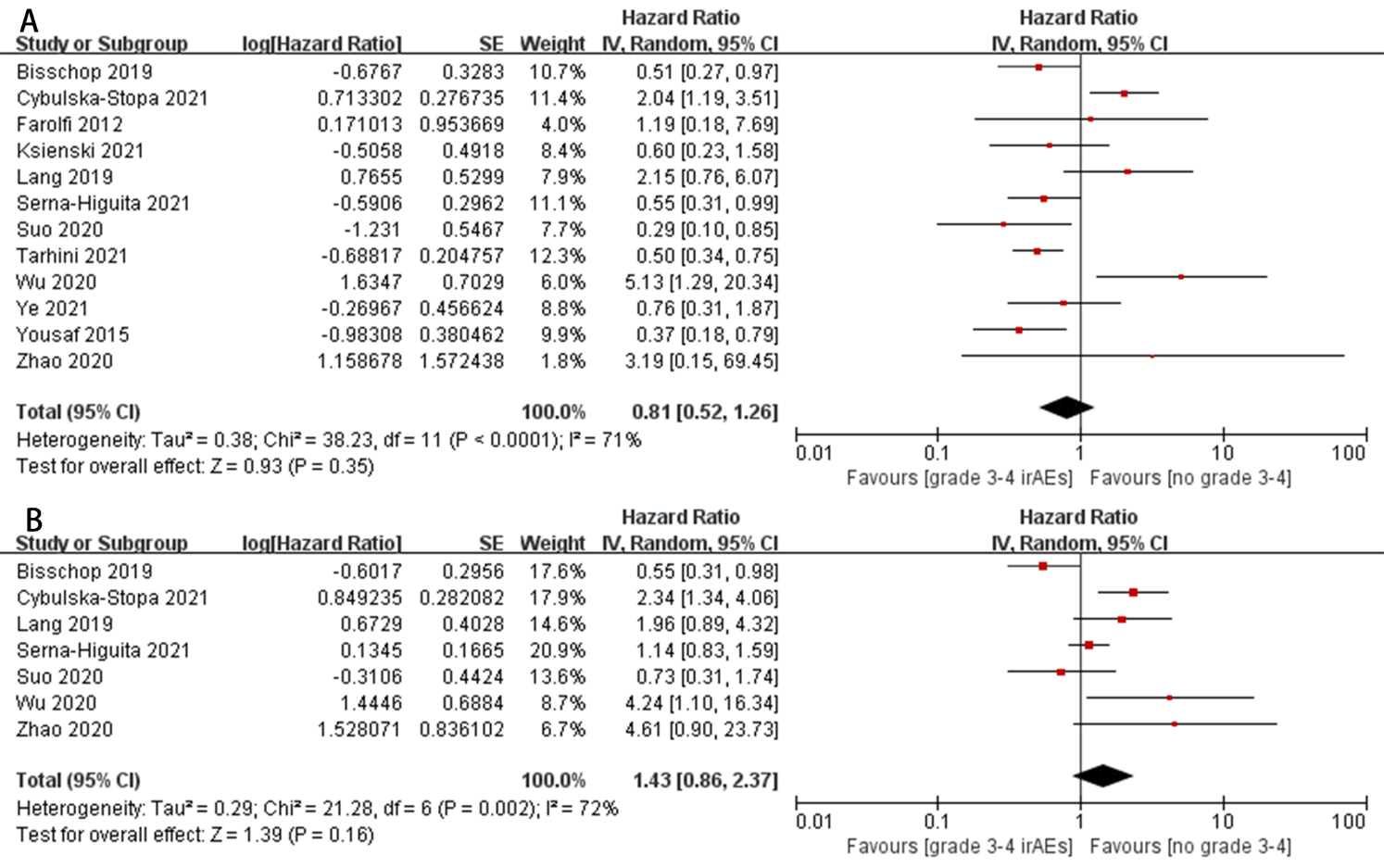


**Supplementary Figure 1.** Forest plots of the association between grade 3-4 irAEs and OS **(A)** and PFS **(B)** in patients with melanoma treated with ICIs.

**
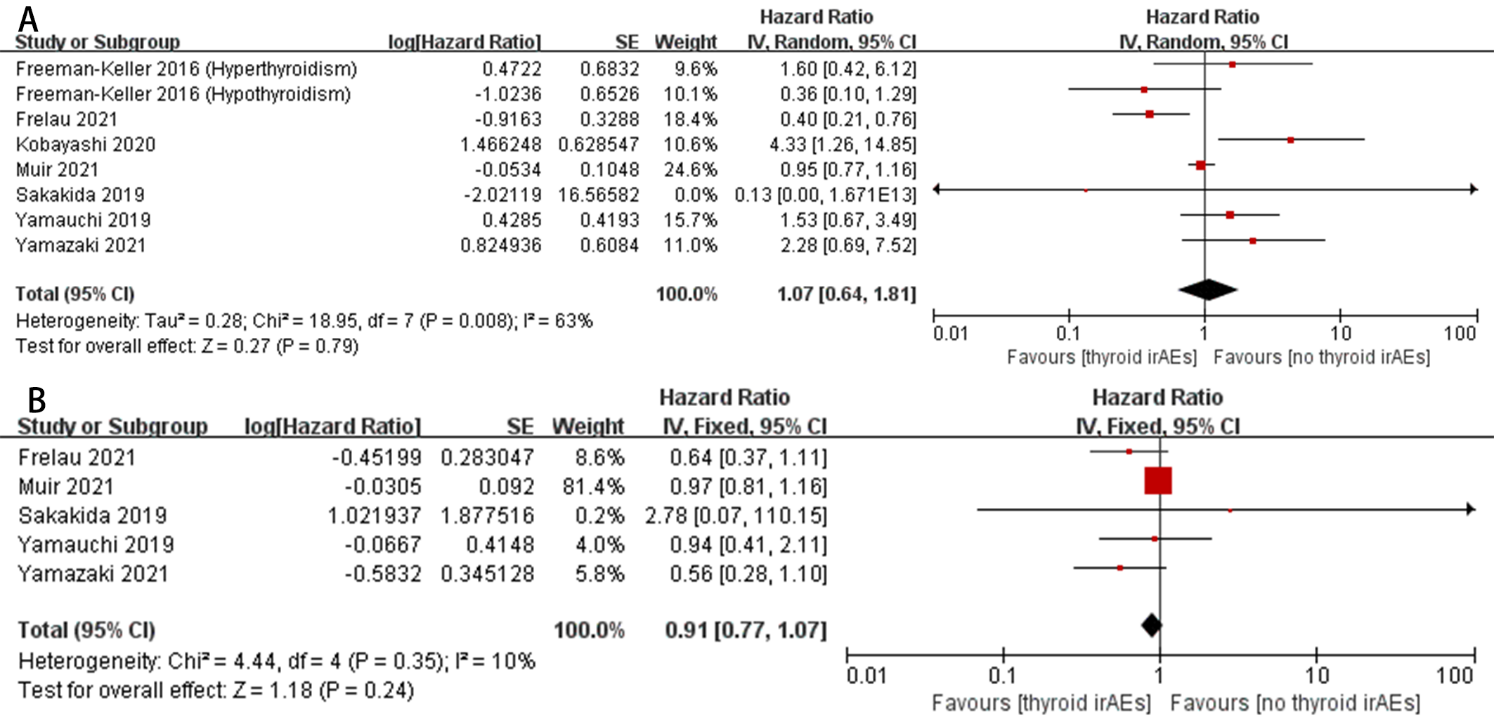
**

**Supplementary Figure 2.** Forest plots of the association between thyroid irAEs and OS **(A)** and PFS **(B)** in patients with melanoma treated with ICIs.


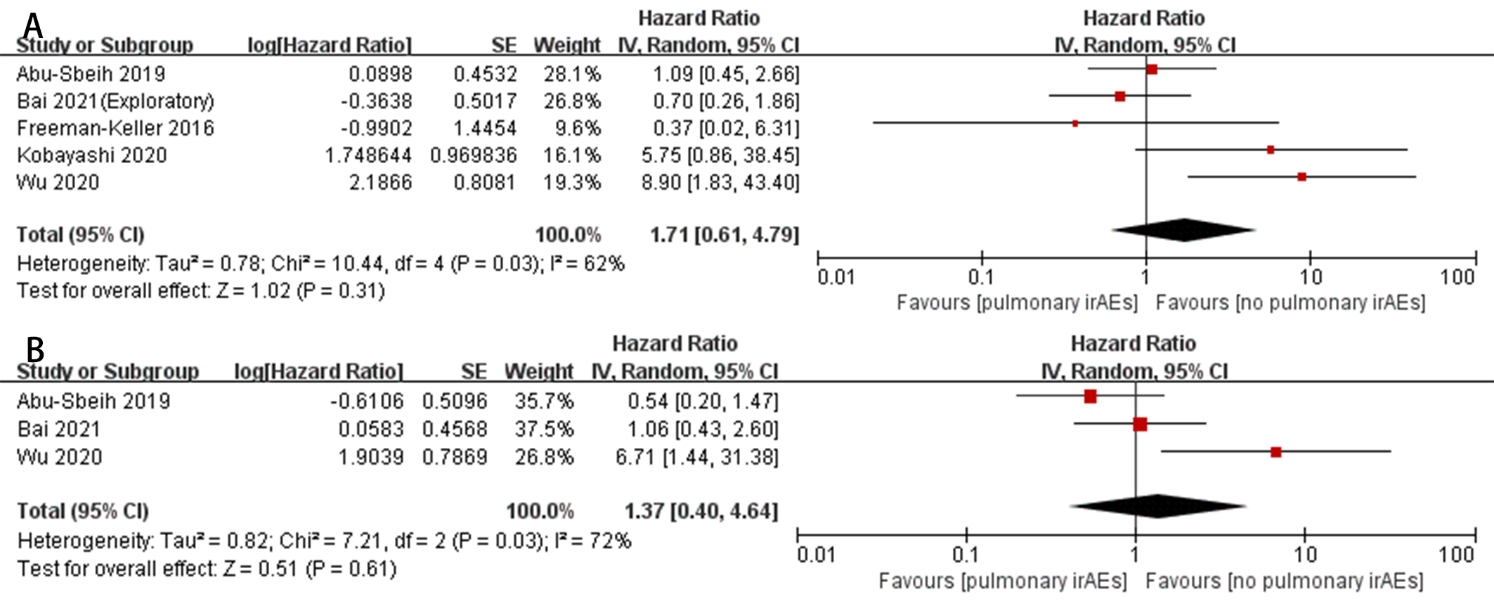


**Supplementary Figure 3.** Forest plots of the association between pulmonary irAEs and OS (A) and PFS (B) in patients with melanoma treated with ICIs.


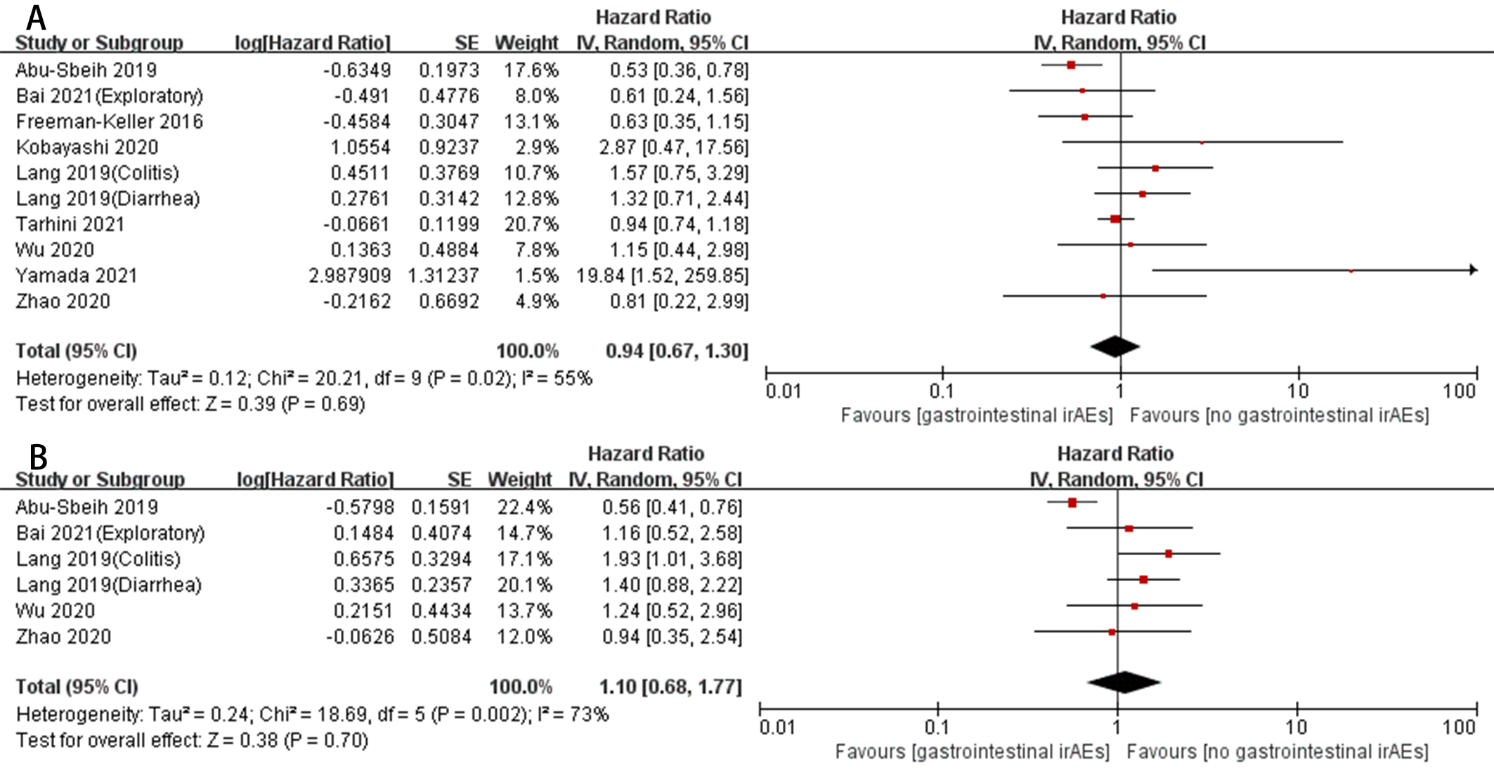


**Supplementary Figure 4.** Forest plots of the association of gastrointestinal irAEs with OS **(A)** and PFS **(B)** in melanoma patients receiving ICIs.


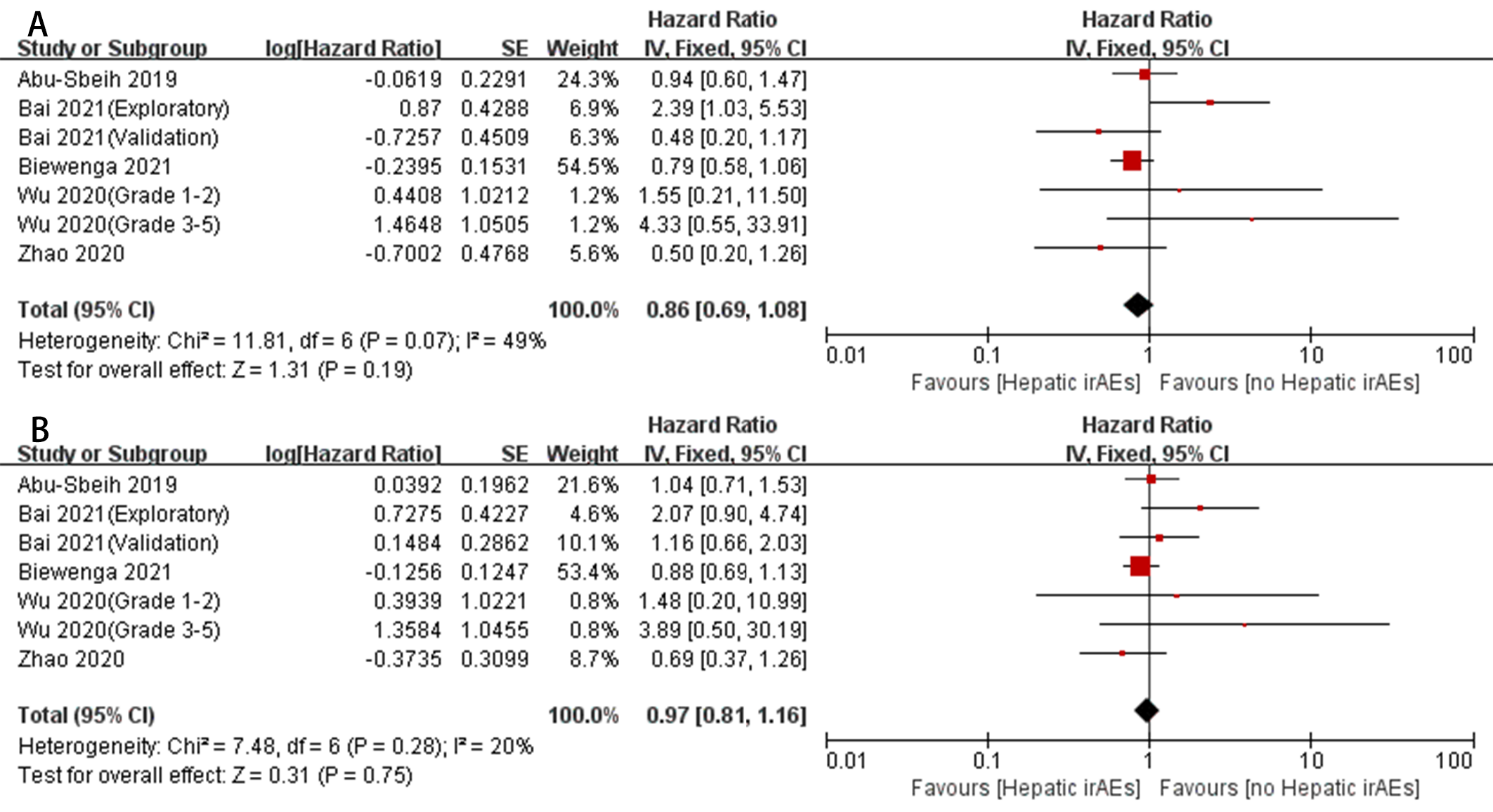


**Supplementary Figure 5.** Forest plots of the association between hepatic irAEs and OS **(A)** and PFS **(B)** in patients with melanoma treated with ICIs.





**Supplementary Figure 6.** Forest plot of the association between any irAEs and OS stratified by study design.





**Supplementary Figure 7.** Forest plot of the association between any irAEs and PFS stratified by study design.





**Supplementary Figure 8.** Forest plot of the association between any irAEs and OS stratified by geographic area.

.



**Supplementary Figure 9.** Forest plot of the association between any irAEs and PFS stratified by geographic area.





**Supplementary Figure 10.** Forest plot of the association between any irAEs and OS stratified by the type of ICIs.





**Supplementary Figure 11.** Forest plot of the association between any irAEs and PFS stratified by the type of ICIs.





**Supplementary Figure 12.** Forest plot of the association between any irAEs and OS stratified by melanoma subtype.





**Supplementary Figure 13.** Forest plot of the association between any irAEs and PFS stratified by melanoma subtype.
